# Cross motor innervation of the hypoglossal nerve—a pilot study of predictors for successful opening of the soft palate

**DOI:** 10.1007/s11325-020-02112-2

**Published:** 2020-06-02

**Authors:** Clemens Heiser, Olivier M. Vanderveken, Günther M. Edenharter, Benedikt Hofauer

**Affiliations:** 1 Department of Otorhinolaryngology, Head and Neck Surgery, Klinikum rechts der Isar, Technische Universität München, Munich, Germany; 2 Translational Neurosciences, Faculty of Medicine and Health Sciences, University of Antwerp, Wilrijk, Antwerp, Belgium; 3 Department of Otorhinolaryngology, Head and Neck Surgery, Antwerp University Hospital, Wilrijkstraat 10, 2650, Edegem, Antwerp, Belgium; 4 Klinikum rechts der Isar der Technischen Universität München, Klinik für Anästhesie, Munich, Germany; 5 Department of Otorhinolaryngology, Head and Neck Surgery University of Freiburg, Freiburg im Breisgau, Germany

**Keywords:** Obstructive sleep apnea, Hypoglossal nerve stimulation, Upper airway stimulation, Cross motor innervation, Hypoglossal nerve

## Abstract

**Purpose:**

Selective hypoglossal nerve stimulation has proven to be a successful treatment option in patients with obstructive sleep apnea. The aim of this pilot study was to investigate if there is a cross-innervation of the hypoglossal nerve in humans and if patients with this phenotype show a different response to hypoglossal nerve stimulation compared to those with ipsilateral-only innervation

**Methods:**

Nineteen patients who previously received a selective hypoglossal nerve stimulation system (Inspire Medical Systems, Golden Valley, USA) were implanted with a nerve integrity system placing electrodes on both sides of the tongue. Tongue motions were recorded one and two months after surgery from transoral and transnasal views. Polysomnography (PSG) was also performed at two months. Electromyogram (EMG) signals and tongue motions after activation were compared with PSG findings.

**Results:**

Cross-innervation showed significant correlation with bilateral tongue movement and bilateral tongue base opening, which were associated with better PSG outcomes.

**Conclusion:**

Cross motor innervation of the hypoglossal nerve occurs in approximately 50% of humans, which is associated with a positive effect on PSG outcomes. Bilateral stimulation of the hypoglossal nerve may be a solution for non-responding patients with pronounced collapse at the soft palate during drug-induced sleep endoscopy.

## Introduction

Breathing cycle-dependent selective hypoglossal nerve stimulation (sHNS) has shown to be a successful treatment option in patients with obstructive sleep apnea (OSA) [1–4]. The hypoglossal nerve (XII) is responsible for all intrinsic and extrinsic muscles of the tongue [5]. XII has a solely motor function. Furthermore, the tongue is a complex muscle network, due to its phenomena to rotate nearly 360° [6]. This network of muscle fibers functions as a hydrostat. Either the muscles have no or only one rigid attachment [7]. The genioglossus muscle is the main opener of the upper airway. It arises from the mental spine of the mandible and inserts in the base of the tongue, without connection to another bone—one of the reasons why the upper airway is so vulnerable to collapse. During wakefulness, these muscle groups help to stiffen the upper airway walls and to prevent healthy subjects’ upper airways from collapsing. The different muscle groups of the tongue can be functionally divided into extrinsic retractors (styloglossus muscle and hyoglossus muscle), extrinsic protrusors (horizontal and oblique part of the genioglossus muscle), and intrinsic stiffeners of the tongue (transverse and vertical muscle, T/V) [5, 6].

The main target muscle for sHNS is the genioglossus muscle (GG) [8]. Its decrease in muscle tone during sleep can lead to obstructions [9]. If GG is activated during sleep by increasing tonic activation, the upper airway can be opened [10, 11]. Contracting the GG fibers pulls the tongue forward from its base and enlarges the upper airway [12]. Until today, not much is known of the complexity of the muscle groups and its innervation of the XII [5, 6, 13]. In 2010 and 2013, the anatomists Mu and Sanders described the innervation of the terminating distal branches of the hypoglossal nerve and the different functions of the muscles groups in a 3D model [5, 6].

In an early pilot study in 2001, Schwartz et al. demonstrated that unilateral stimulation of the hypoglossal nerve is feasible and a potential option for OSA patients [14]. In multicenter international trials and registers, sHNS showed efficacy, safety, and effectivenss in a clinical routine [15]. It is known for a while that different postoperative tongue motions are associated with heterogeneous therapy outcomes [12, 16]. The crucial part for the different tongue motions is the selective stimulation lead placement during the surgical procedure. A new surgical classification system of the terminating fibers of the XII was developed accordingly [17]. Another important point for improving clinical outcome is the opening of the soft palate during stimulation [18]. For this reason, tongue motions and its related correct selective stimulation of the XII fibers are one of the key steps for success [16].

The aim of this pilot study was to investigate if there is a cross-innervation of the XII in humans and if patients with this phenotype show a different response to sHNS compared to those with ipsilateral-only innervation.

## Materials and methods

The study was approved by ethics committees and registered with http://www.clinicaltrials.gov (NCT02907398). All procedures followed were in accordance with the ethical standards of the responsible committee on human experimentation (institutional and national) and with the Helsinki Declaration of 1964 and later revision. Informed consent was signed by every patient. Consecutive patients, who received an sHNS implant with previously described inclusion criteria (Inspire II, Inspire Medical Systems, Maple Grove, MN, USA) at a single center in Munich, Germany, from October 2017 to November 2018 were included [3].

### Intraoperative neuromonitoring

Intraoperative neuromonitoring was performed using a nerve integrity monitoring system (NIM 3.0, Medtronic Xomed, Jacksonville, FL, USA). The surgical steps have been previously described in detail [19–21] Furthermore, short-acting muscle relaxants were used for anesthesia to accommodate intraoperative examination of tongue stimulation in response to electrical stimulation. Four single-use 18-mm Prass Paired EMG-(electromyography) electrodes (Medtronic Xomed, Ref 8227304) were placed in the tongue: two on the right side and two on the contralateral left side [21].

This EMG was needed to distinguish between the main retractors (hyoglossus and styloglossus muscle) and main protrusors (horizontal and oblique genioglossus muscle). The electrodes for the retractors were placed 5 cm from the tip of the tongue just below the lateral corner of the tongue, superficially below the mucosa membrane on the left and right sides. The electrode for the protrusors were placed paramedian in the mouth floor on the left and right side.

During surgery, a bipolar electrical stimulation probe was used (Xomed Side-by-Side Bipolar Stimulating Probe, Medtronic Xomed, Ref 8225401) to differentiate the various nerve fibers [21]. After placement of the stimulation lead, the sHNS system was tested with an amplitude of 0.5 V (pulse width of 90 μs and a frequency of 33 Hz).

The EMG activity was recorded in all four channels with focus on the amplitudes:

1. genioglossus channel right, 2. genioglossus channel left, 3. hyoglossus channel right, and 4. hyoglossus channel left. The EMG signals were saved and later analyzed by a blinded person. For a cross over innervation of the XII, a maximum EMG amplitude difference of 1:10 was accepted. Larger ratios (> 1:10) were considered as ipsilateral innervations of the hypoglossal nerve.

Tongue motions were recorded at different time points:

- One month (M1) after surgery during the first activation of the system
- Two months (M2) after surgery during an in-lab polysomnography

Different tongue motions were recorded as described before [12]:

- Bilateral protrusion (BP)
- Right protrusion (RP)
- Mixed activation (MA)

Also, an awake transnasal flexible endoscopy was performed at M1 and M2 to assess the opening of the tongue base and soft palate during stimulation. At M1 and M2, the amplitude for stimulation of the functional threshold was used. The functional threshold is defined as the level of voltage at which bulk tongue motion is achieved [12]. At M2, the titrated amplitude for stimulation from the in-lab PSG was used.

Furthermore, the degree of palatoglossus coupling (PGC) was calculated as described before [18]: 0 = no change, 1 = some enlargement, and 2 = large enlargement.

### Statistical analysis

Version 23.0 of the Statistical Package for the Social Sciences (SPSS) software (IBM, Armonk, NY) was used. Descriptive statistics were calculated for demographic variables. A paired t test was used to compare baseline and postimplantation values. The patients’ data were compared using the Mann–Whitney U test for metric data. Spearman rank correlation coefficient (*R*) was used for the analysis of correlations (0.00–0.19 = very weak correlation, 0.20–0.39 = weak correlation, 0.4–0.59 = moderate correlation, 0.60–0.79 = strong correlation, 0.80–1.00 = very strong correlation). Data are given as mean ± standard deviation if not otherwise stated. *P* values < 0.05 were considered statistically significant.

#### Ethical considerations

Informed consent was obtained for each patient. The study was approved by the local ethic committee (Faculty for Medicine, ethical committee, Technical University of Munich, Germany).

#### Data availability statement

All relevant data are within the paper.

#### Results

Between October, 2017 and November, 2018, 19 patients, who received an implant, were enrolled. The demographic baseline characteristics are shown in Table 1.

**Table 1:** Baseline characteristics of the patients who received a selective upper airway stimulation. *AHI* apnea–hypopnea index, *p < 0.001 vs. baseline

**Demographics**	
*Number of patients*	19
*Age (in years)*	59.4 ± 11.2
*Gender*	Male: *n* = 18 (95%)
	Female: *n* = 1 (5%)
*Body mass index (BMI), kg/m*^*2*^	29.8 ± 3.9
*AHI, events/h (baseline)*	43.04 ± 16.19
AHI, events/h (month 2)*	6.7 ± 21.2*

The measuring of the EMG amplitudes during stimulation of the right hypoglossal nerve, revealed in 9 (47%) patients a cross over (bilateral) innervation of the genioglossus muscle. Ten (53%) showed an ipsilateral innervation. Further details for every patient are shown in Table 2.

**Table 2:** Amplitudes of the nerve integrity monitoring system for the genioglossus muscle to measure cross over innervation from right to left sides

***Patient N°***	**NIM amplitude GG right**	**NIM amplitude GG left**	***Ratio GG right***: ***GG left***
*1*	368	214	*1:1.7*
*2*	138	16	*1:8.6*
*3*	31,590	302	*1:104.6*
*4*	1125	42	*1:26.7*
*5*	179	7	*1:25.5*
*6*	742	9	*1:82.4*
*7*	896	701	*1:1.2*
*8*	1237	10	*1:123.7*
*9*	173	66	*1:2.6*
*10*	242	48	*1:5*
*11*	959	18	*1:53.2*
*12*	1626	186	*1:8.7*
*13*	1798	32	*1:56.1*
*14*	2147	237	*1:9*
*15*	2056	88	*1:23.3*
*16*	20,544	770	*1:26.6*
*17*	237	82	*1:2.8*
*18*	3763	47	*1:80*
*19*	599	82	*1:7.3*
*Average (bi)*	714	181	1:3.9
*SD (bi)*	718	210	1:3.4
*Average (ip)*	6399	133	1:48.2
*SD (ip)*	10,731	241	1:44.5

For a cross over innervation of the hypoglossal nerve, a maximum EMG amplitude difference of 1:10 was accepted. Bigger ratios (> 1:10) were considered as ipsilateral innervations of the hypoglossal nerve. The gray marked cells are patients with bilateral innervation of the hypoglossal nerve.

*NIM* nerve integrity monitoring, *GG* genioglossus muscle, *N* number, *SD* standard deviation, *bi* bilateral innervation, *ip* ipsilateral innervation

**Fig. 1:**
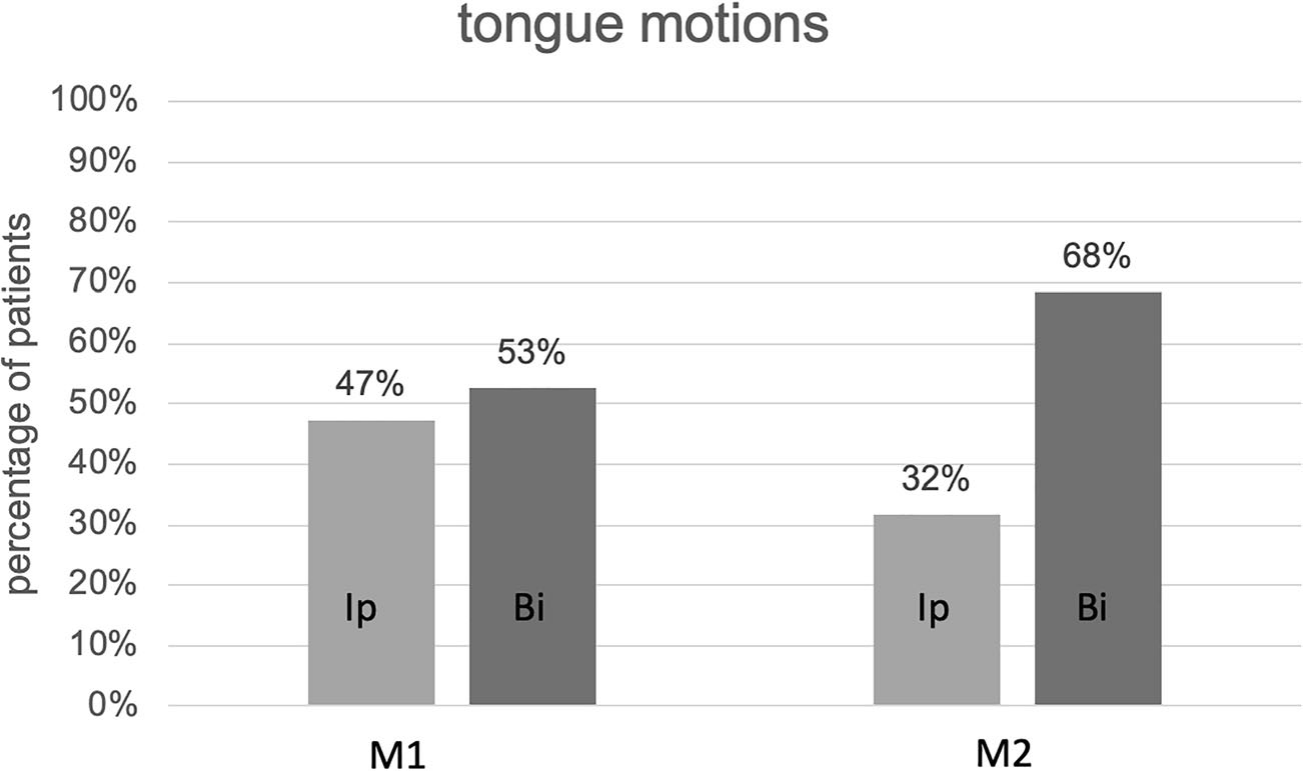
The percentage of patients with tongue motions at months one and two after implantation of the system. *Bi* bilateral protrusion, *Rp* right protrusion, *M1* month 1, *M2* month 2

Nine patients showed a right protrusion at M1 and 6 patients at M2. The other 11 patients at M1 had a bilateral protrusion, meanwhile at M2, 13 patients revealed a bilateral tongue motion **(**Fig. 1**)**. No mixed activations were recorded.

**Fig. 2:**
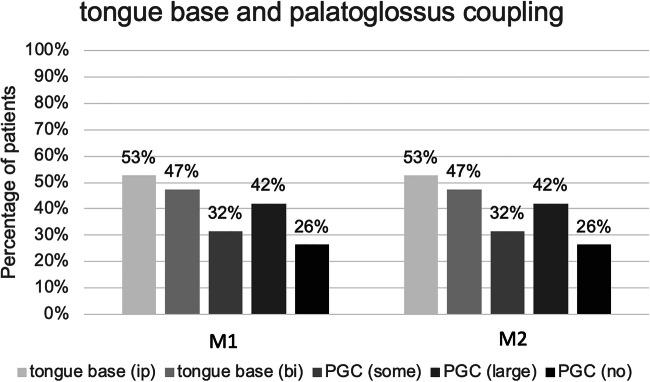
The percentage of patients with motions at the tongue base and calculated PGC effect at the retropalatal region at months one and two after implantation of the system. PGC effect was calculated and coded with: *no* = no enlargement at the soft palate, *some* = some enlargement at the soft palate, and *large* = marked enlargement at the soft palate.*bi* bilateral protrusion, *ip* ipsilateral protrusion, *PGC* palatoglossus coupling, *M1* month 1, *M2* month 2

Almost 50% of the patients showed an ispilateral or bilateral opening of the tongue base during stimulation at M1 and M2 **(**Fig. 2**).** The calculated PGC effect was seen in more than 74% at M1 and M2. These 74% of patients had at least some enlargement at the level of the soft palate during stimulation.

Overall, the baseline (pre-implant) total and titrated apnea–hypopnea index (AHI) of all patients was reduced from 43.04 ± 16.19/h to 19.51 ± 17.59/h (total AHI at M2; *p* < 0.001) and to 6.7 ± 21.2/h (titrated AHI at M2; *p* < 0.001). The group of patients with bilateral innervation of the hypoglossal nerve (cross-innervation) showed a reduction in AHI from 39.07 ± 18.74/h to 15.98 ± 10.39/h (total AHI at M2; *p* < 0.005) and to 5.3 ± 28.1/h (titrated AHI at M2; *p* < 0.001). The ispilateral innervation group showed a reduction from 45.26 ± 13.57/h to 22.98 ± 23.67/h (total AHI at M2; *p* < 0.023) and to 7.0 ± 22.1/h (titrated AHI at M2; *p* < 0.001; Table 3). Further parameters for the apnea index (AI), hypopnea index (HI), oxygen disturbance index (ODI), minimum SpO_2_, and mean SpO_2_ are shown in detail in Table 3.

**Table 3:** Breathing parameters during PSG before and after implantation

	Total			Bilateral innervation			Ipsilateral innervation		
	Baseline	M2	*p* value	Baseline	M2	*p* value	Baseline	M2	*p* value
Titrated AHI (n/h) ± SD	43.04 ± 16.19	6.7 ± 21.2	< 0.001	39.07 ± 18.74	5.3 ± 28.1	< 0.001	45.26 ± 13.57	7.0 ± 22.1	< 0.001
AHI (n/h) ± SD	43.04 ± 16.19	19.51 ± 17.59	< 0.001	39.07 ± 18.74	15.98 ± 10.39	0.005	45.26 ± 13.57	22.98 ± 23.67	0.023
AI (n/h) ± SD	15.35 ± 14.56	8.85 ± 14.49	0.116	12.44 ± 9.81	7.00 ± 9.55	0.010	18.01 ± 19.04	11.38 ± 19.11	0.451
HI (n/h) ± SD	27.68 ± 14.03	10.82 ± 6.56	< 0.001	26.63 ± 16.16	9.30 ± 4.79	0.021	27.22 ± 12.54	11.59 ± 8.06	0.021
ODI (n/h) ± SD	43.53 ± 16.65	20.89 ± 17.36	< 0.001	39.00 ± 17.92	17.33 ± 10.68	0.005	45.11 ± 13.75	23.63 ± 23.11	< 0.001
Min SpO_2_ (%) ± SD	78.89 ± 5.97	84.63 ± 4.70	< 0.001	78.89 ± 4.08	86.44 ± 3.36	< 0.001	79.78 ± 7.41	83.67 ± 5.15	0.094
Mean SpO_2_ ± SD	93.53 ± 1.95	93.32 ± 1.38	0.578	93.89 ± 1.05	93.89 ± 0.93	1.000	93.89 ± 1.36	93.11 ± 1.27	0.211

*AHI* apnea–hypopnea index, *SD* standard deviation, *bi* bilateral innervation, *ip* ipsilateral innervation, *M2* month 2, *AI* apnea index, *HI* hypopnea index, *ODI* oxygen disturbance index, *min SpO*_*2*_ minimum oxygen saturation, *mean SpO*_*2*_ mean oxygen saturation

**Fig. 3:**
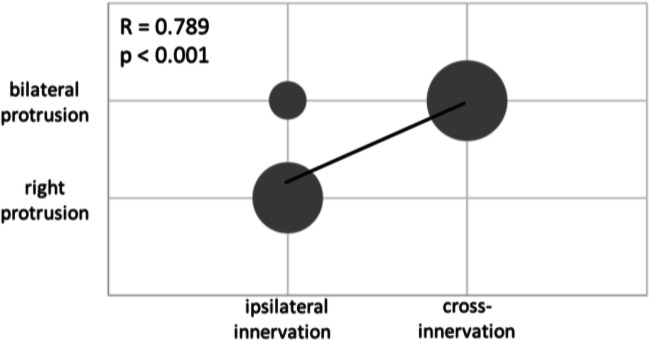
Correlation between tongue motion and innervation of the hypoglossal nerve.The bubble plot illustrates the correlation of the type of innervation and the tongue motion at M2. A strong correlation between a cross-innervation and a bilateral tongue protrusion was detected

The cross-innervation showed a significant correlation with a bilateral tongue movement at M1 and M2 (*R* = 0.798, *p* < 0.001; *R* = 0.620, *p* = 0.006; Fig. 3). Furthermore, a significant correlation could be found between the bilateral innervation and bilateral tongue base opening at M1 and M2 (*R* = 0.671, *p* = 0.002; *R* = 0.671, *p* = 0.002). Additionally, patients with cross-innervation had a greater PGC effect than patients with ipsilateral innervation (*R* = 0.707, *p* = 0.006; *R* = 0.707, *p* = 0.001). These findings imply that patients with cross-innervation tend to have a bilateral tongue protrusion, hence a better opening of the airways at the level of the tongue and consequently a better PCG effect.

At the same time, PGC shows a significant correlation with the relative reduction of AHI at M2 (*R* = 0.482, *p* = 0.037). Cross-innervation of the hypoglossal nerve seems to be associated with a better PGC and reduction of AHI.

## Discussion

Our pilot study confirmed the efficacy of sHNS in OSA patients as it has been described before [2, 22–24]. The highly selective placement of the stimulation lead and the associated correct activation of the muscle groups help to prevent the upper airway from collapsing and support an active opening. Therefore, the right nerve fibers have to be the target [21]. It is essential to exclude the lateral fibers of the XII. The main objective during surgery is to distinguish between the lateral and medial nerve fibers [19]. In our pilot study, the electrodes for neuromonitoring were not only on one side as usual in clinical routine, but identically on both sides of the tongue. This two-sided electrode placement supports the measurement of a contralateral innervation of the XII by recording the opposite EMG. Preliminary results have shown that a bilateral innervation of the XII seems to be concordant with a better opening on the soft palate [18]. This type of innervation could appear to be a key factor during HNS. Safirruddin et al. described variations in the opening of the retropalatal region during stimulation among patients [18, 25]. In all patients, regardless of therapy response, retrolingual opening during stimulation was present [18, 25]. But there is a difference in the opening of the soft palate between patients. One hypothesis from earlier studies to describe this effect on the soft palate is the palatoglossus coupling (PCG). The palatoglossus muscle is a thin muscle running in the anterior pillar from the soft palate into the tongue [18, 26, 27]. If during stimulation, the tongue is flattened and elongated in a stiffened manner; this muscle acts like an anchor and pulls the soft palate forward. The intrinsic transverse and vertical muscle fibers need to be activated to stiffen the tongue. The palatoglossus muscle exhibits activation to negative pressure in patients with OSA, which could play a role in restoring upper airway patency [27]. In such cases, the soft palate may have the ability to maintain patency during respiration, but the falling back of the mass of the tongue creates secondary palatal collapse in the presence of a primary base-of-tongue collapse etiology. This may be explanatory for how the stiffened, unhindered protrusion during stimulation helps to decrease or eliminate collapse of the soft palate. One hypothesis why OSA patients with sHNS are not fully responding to therapy is the persisting of obstructions at the soft palate [25, 28]. Even in some patients, a protrusion of the stiffened tongue can be detected. If a bilateral stimulation of the XII is performed, an activation of the intrinsic T/V muscles probably shows a stronger effect on the soft palate. This could be related in pulling both palatoglossus muscles forward. An anchor on both sides tying the soft palate to the tongue produce a stronger effect on the palate opening, thereby overcoming the vulnerability of the Starling resistor and Bernoulli effect. This hypothesis could be the explanation why a better clinical outcome in our sHNS patients could be detected with cross over innervation of the XII. Also, in patients with a measured intraoperatively ipsilateral innervation and initially confirmed mismatch of obstructions at the palate, who are not fully responding to therapy, a bilateral stimulation of the XII could potentially solve the problem.

Currently, there is no known predictor for whether any given patient will display a bilateral versus ipsilateral phenotype, and even with an ipsilateral innervation, it is not certain that these patients will fare worse as many are full-therapy responders. Further research is needed to distinguish preoperatively the instances in which bilateral stimulation of the XII could be clinically successful and warranted in the setting of the initial implant. Likewise, sub-optimal therapy responders with clear ipsilateral innervation-activation phenotype could be “upgraded” to bilateral stimulation to further this line of inquiry. The development of new stimulation systems may also include the development of bilateral stimulation strategies, for which patients with pronounced collapse at the soft palate during drug-induced sleep endoscopy might be good candidates. This preliminary data will help to set up further clinical trials on the existence and predictive value of cross motor innervation. At the moment, patients with a complete concentric collapse at the soft palate are excluded from sHNS. Probably these patients could still be responders for HNS, if cross motor innervation is presented.

Furthermore, another question would be, if a patient who shows a complete concentric collapse during drug-induced sleep endoscopy—an exclusion criterion for sHNS—could this patient even be a good responder to therapy due to a cross motor innervation of the hypoglossal nerve? [28] Or should he/she go one step further, which would mean to do a bilateral stimulation of the hypoglossal nerve to solve the obstructions at the soft palate? Further research is needed to answer these questions.

## Conclusion

Based on the results of this pilot study, cross motor innervation of the hypoglossal nerve seems to appear in about 50% of humans. This bilateral innervation seems to have a more positive outcome on the soft palate during stimulation and seems to be associated with a better impact on the clinical outcome. A bilateral stimulation of the hypoglossal nerve could be a solution for non-responders at the level of the soft palate.
